# The Prevalence of Arrhythmias, Including Premature Supraventricular and Ventricular Beats and Other Electrocardiographic Patterns, in 24-Hour Holter Monitoring in Patients with Overweight and Obesity

**DOI:** 10.3390/life14091140

**Published:** 2024-09-09

**Authors:** Irena Anna Dykiert, Krzysztof Kraik, Lidia Jurczenko, Paweł Gać, Rafał Poręba, Małgorzata Poręba

**Affiliations:** 1 Department of Physiology and Pathophysiology, Division of Pathophysiology, Wroclaw Medical University, 50-368 Wrocław, Poland; 2 Students’ Scientific Association of Cardiovascular Diseases Prevention, Wroclaw Medical University, 50-368 Wrocław, Poland; 3 Department of Environmental Health, Occupational Medicine and Epidemiology, Wroclaw Medical University, 50-345 Wrocław, Poland; 4 Department and Clinic of Angiology and Internal Medicine, Wroclaw Medical University, 50-556 Wrocław, Poland; 5 Department of Paralympic Sport, Wroclaw University of Health and Sport Sciences, 51-617 Wrocław, Poland

**Keywords:** electrocardiography, body mass index, premature ventricular beats, premature supraventricular beats, waist–hip ratio

## Abstract

Objectives: this study aims to evaluate the prevalence of various arrhythmias and other electrocardiographic patterns within the group of individuals with overweight and obesity. Methods: One hundred eighty-one adults (90 females and 91 males) were qualified for inclusion in the experimental group. All participants had a body mass index (BMI) exceeding 25 kg/m^2^ (98 patients with obesity and 83 with overweight). The mean BMI in the obesity group was 33.6 kg/m^2^, and all participants had class 1 obesity. The control group comprised 69 individuals (56 females and 13 males) with normal BMI. The basic measurements were performed, and the participants filled out questionnaires describing their health conditions and lifestyles. Each participant underwent an electrocardiographic (ECG) examination and a 24 h Holter ECG examination. Results: In patients with class 1 obesity compared to the control patients, the average numbers of premature ventricular beats (PVBs) and premature supraventricular beats (SPBs) were statistically significantly higher (*p =* 0.030 and *p =* 0.042). There was a positive correlation between body weight and PVB (*p =* 0.028) and between body weight and SPB (*p =* 0.028). Moreover, BMI and waist circumference were correlated with SPB (*p =* 0.043 and *p =* 0.031). In the backward stepwise multivariate regression model considering 24 h Holter ECG monitoring, concerning SPB as the dependent variable, it was observed that BMI (especially obesity class 1), type 2 diabetes, and thyroid disease exhibited the highest regression coefficients. Conclusions: obesity, even in class 1, might be a factor in a more frequent occurrence of abnormalities in electrocardiographic tests.

## 1. Introduction

In recent decades, the global prevalence of obesity and overweightness has reached pandemic proportions, giving rise to a myriad of health concerns. According to the World Health Organization (WHO), in 2022 [1], the global population of individuals with overweight and obesity reached 2.5 billion. Approximately 43% of all adults worldwide were classified as overweight and 16% as obese. An even more alarming fact is that the number of individuals with obesity nearly doubled between 1990 and 2022. Eurostat reported that 53% of the combined adult population of the EU, Norway, Serbia, and Turkey had BMI > 25 kg/m^2^. Moreover, according to data from a European health interview survey, more men than women were overweight in each country. There was also a noticeable relationship between an increase in the percentage of the overweight population and age [2]. According to data published in 2021 by GUS (Polish Central Office of Statistics), in Poland, in 2019, 57% of adults had excessive body weight (overweight or obesity), whereas in 2014, it was slightly over 53%. Over the past few years, the percentage of adults with obesity in Poland has been steadily increasing [3]. It has been estimated that by 2025, in Poland, 26% of women and 30% of men will be classified as obese.

One particularly concerning aspect is the intricate relationship between excessive body weight and the development of cardiac arrhythmias. Heart rhythm is usually characterized by abnormal heart rate and has attracted considerable attention due to its potential relationship with cardiovascular events. As a consequence of the obesity epidemic, understanding the underlying mechanisms that link obesity and arrhythmias has become imperative in the realm of both clinical and research endeavors. To date, there have been studies addressing the issue of arrhythmia in individuals with overweight and obesity. Mainly, the currently available literature on this topic suggests an increased prevalence of arrhythmia among these patients, especially in the form of atrial fibrillation (AF) [4]. Other available studies have revealed similar results. They also describe the relationship between the incidence of AF and weight loss [5,6,7].

Noting that excessive weight has emerged as a significant global health issue and some reports have indicated that being overweight and obese could predispose individuals to arrhythmia, this study aims to evaluate the prevalence of various arrhythmias and other electrocardiographic patterns within the group of individuals with overweight and obesity. 

## 2. Materials and Methods

### 2.1. Study Population

This study was conducted at the Department of Pathophysiology of Wroclaw Medical University from 2020 to 2023. Ethical consent for this study was obtained from the Wroclaw Medical University Ethics Committee, ensuring adherence to the principles of Good Clinical Practice and the Declaration of Helsinki.

**Figure A1:**
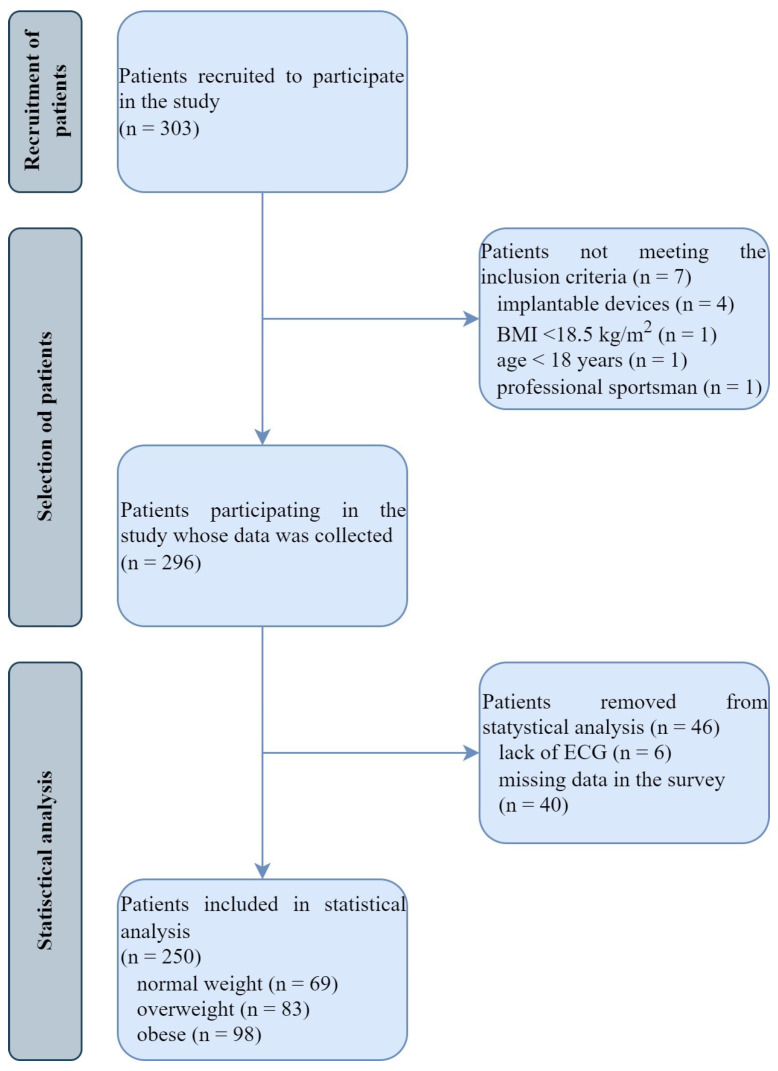
Flowchart presenting the selection of participants.

A total of 181 adults (90 females and 91 males) of the Caucasian race were qualified for the experimental group. The patients were selected from the population of Wroclaw and the nearby regions of Lower Silesia, Poland. Studies were carried out in the Department of Pathophysiology, and all participants were volunteers who had previously participated in other studies in the department. They were recruited when they met the inclusion criteria and signed the informed consent form after accepting the study rules. No payment or incentive was offered for participation. The exclusion criteria were age below 18 years, BMI below 18.5 kg/m^2^, presence of implantable devices, and being a professional athlete. Moreover, the participants whose ECG was missing or whose questionnaires were filled out incompletely were excluded from this study. All these participants had a BMI exceeding 25 kg/m^2^ (98 patients had obesity, and 83 were overweight). The control group comprised 69 individuals (56 females and 13 males) with normal BMI—the characteristics of the study and control groups are presented in Table 1. Appendix A includes a flowchart illustrating the selection of participants (Figure A1).

### 2.2. Trial Design

The participants filled out the informed consent form to participate in this study and were subsequently provided with a detailed description of the procedures. In the initial phase of the research, the participants were asked to fill out a specialized questionnaire, including inquiries about various domains of their lifestyle, such as physical activity, the consumption of alcohol and other stimulants, smoking, dietary patterns, medical conditions, family medical history and psychological background. None of the participants declared in the questionnaire a personal and/or family history of genetic rhythm disorders. Additionally, none of them stated an incidence of illicit drug use, as well as any other stimulants except for coffee, which was used in small quantities, 1–3 cups per day. BMI was calculated by dividing body weight in kilograms by the square of height in meters. WHR (waist-to-hip ratio) was calculated by dividing waist circumference by hip circumference in centimeters. Both measurements adhered to the guidelines specified in the WHO protocol [8,9]. The 12-lead ECG and 24 h Holter ECG examination were performed in the next part of the study. Twenty-four-hour Holter ECG was performed on a typical day for participants to ensure that the collected data were reliable and that they were not affected by unusual behavior from the participants.

The ECG was recorded and analyzed at a speed of 25 mm/s. At first, ECGs were analyzed by two medicine students trained in electrocardiography and verified by two cardiologist members of the study team. Twenty-four-hour Holter ECG analysis was examined by the cardiologist-in-training and then verified by the skilled cardiologist. The values of each parameter were presented as the maximum, minimum, and mean values with SD. Additionally, this study was extended to include the RR interval variability, that is, time-domain heart rate variability analysis according to the classic standards by the Task Force of the European Society of Cardiology and the North American Society of Pacing Electrophysiology [10].

### 2.3. Statistical Analysis

A statistical analysis was conducted using the licensed CSP “STATISTICA 13” (StatSoft Poland, Kraków, Poland). For quantitative variables, arithmetic means (x) and standard deviations (SD) were calculated for the parameters recorded in the studied groups. The distribution of variables was tested with the W Shapiro–Wilk test. In the case of the quantitative variables of normal distribution, a further statistical analysis used the univariate ANOVA variance analysis. The ANOVA Kruskal–Wallis analysis was also used for non-normally distributed and quantitative variables. Statistically significant differences between the arithmetic means were determined by the post hoc Newman–Keuls test. Results for qualitative variables were expressed in the form of percentage lists. The chi-squared test of maximum likelihood was used in further statistical analysis for qualitative variables. Correlation and regression analyses were performed to determine the relationship between the studied variables. In the case of variables with normal distribution, the Pearson r correlation coefficients were determined; in the case of variables not normally distributed, the Spearman r coefficients were established. Regression analysis was performed using the multivariate backward stepwise method. The parameters of models obtained in the regression analysis were estimated using the technique of least squares. Results at *p* < 0.05 were assumed to be statistically significant.

## 3. Results

There were statistically more arterial hypertension patients in the group of individuals with obesity and overweight in comparison to the control group, as well as more type 2 diabetes cases in patients with obesity than in the control group. All characteristics of the entire study group and its subgroups are presented in Table 1.

During the 24 h Holter ECG monitoring, the mean heart rate (HR) was 72.73 ± 0.55 bpm, with a minimum of 53.93 ± 0.50 bpm and a maximum of 117.33 ± 1.19 bpm. Instances of bradycardia (27.94 ± 9.15 with a mean of 38.39 ± 4.37 bpm) and tachycardia (23.02 ± 12.70 with 149.16 ± 20.39 bpm) were observed. The occurrence of premature ventricular and supraventricular beats was as follows: PVBs (premature ventricular beats) 308.39 ± 88.75 and SPBs (premature supraventricular beats) 374.10 ± 129.18. Then, the variability of the RR interval and time-domain heart rate was analyzed. The full results are summarized in Table 2 and Table 3.

**Figure 1:**
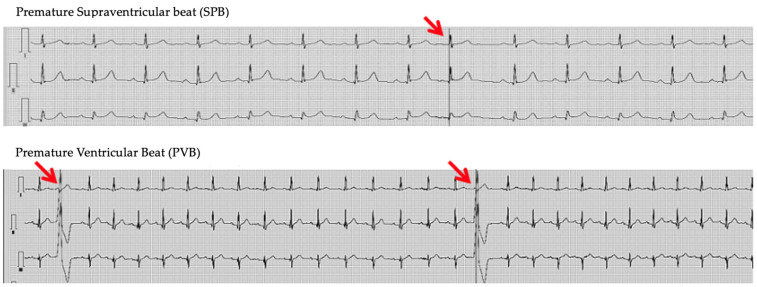
Typical ECG recordings of SPB and PVB found in our study group (indicated by the arrow).

In the 24 h Holter ECG monitoring within the study subgroups (A, B, C), statistical significance was observed in the incidence of PVB and SPB, with these findings being significantly more common in the obese group when compared with the control group. No other significant differences in 24 h Holter ECG parameters, indicating irregular rhythm, were observed between the groups. The prevalence of atrial fibrillation in both the overweight and obesity groups was slightly higher than in the control group (where it was 0%). However, atrial differences are not significant. Tachycardia incidence in the obese and overweight groups was higher than in the control individuals; however, the difference did not reach significance. Figure 1 contains representative ECG recordings of SPB and PVB.

Table 2 and Table 4 summarize the 24 h Holter ECG monitoring parameters for the entire study group and its subgroups. 

In HRV analysis, only mRR in 24 h monitoring and mRR during daily activity were significantly higher in the obese and overweight subgroups than in the control subgroup. As for SDNN, the known time-domain parameter, there was only a trend toward lower values in the overweight subgroup compared to the controls; however, it did not reach statistical significance. 

No statistically significant correlations were found between the study group’s body mass parameters and time-domain heart rate variability parameters. The results of this analysis are presented in Table 5.

The were correlations between body weight and the occurrence of PVB (r = 0.14, *p* = 0.028) and SPB (r = 0.14, *p* = 0.028). Furthermore, both BMI and waist circumference were found to be positively correlated with SPB. The linear relationship found in the current study is presented in Table 6.

**Figure A2:**
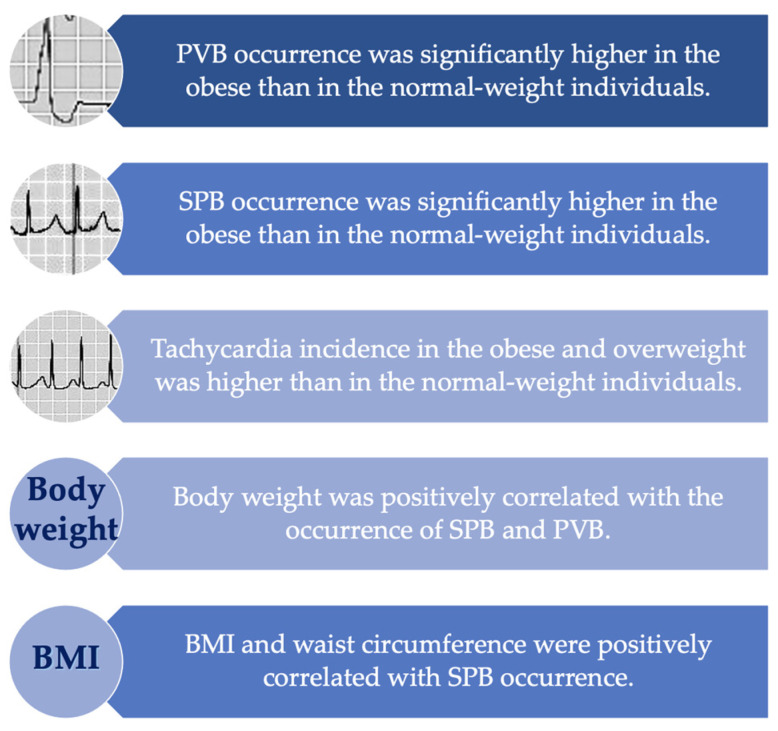
Key findings of this study.

Regression analysis assessed the importance of other variables and co-occurring conditions for the linear relationships shown in the correlation analysis. Models were estimated for PVB and SPB as dependent variables using anthropometric parameters, comorbidities, and smoking as potential independent variables. The estimation was made using backward stepwise multivariate analysis. A statistically significant model was obtained for SPB: SPB = 82.292 BMI + 791.956 type 2 diabetes + 918.975 thyroid disease. It was observed that higher BMI, type 2 diabetes, and thyroid disease are independently associated with higher SPBs. The full results are summarized in Table 7. Appendix A includes a chart presenting this study’s key findings (Figure A2).

## 4. Discussion

Obesity is the excessive accumulation of body fat, which can result in health problems [1]. Visceral abdominal fat poses a greater health risk compared to total body fat. There are many indicators proposed for patient evaluation, such as body mass index (BMI), waist circumference (WC), waist-to-hip ratio (WHR), waist-to-height ratio (WHtR), fat mass index (FMI), fat-free mass index (FFMI), and fat mass body percentage (FM%). Body mass index has been the standard measure used to classify obesity for years and is still widely utilized for its feasibility and non-invasive character. Nevertheless, none of the above indices individually was an effective cardiovascular predictor [11]. 

A holistic approach allows for the more efficient treatment of complex diseases such as obesity. Patients with obesity are in the risk group for many cardiovascular and noncardiovascular diseases. Due to obesity’s impact on the cardiovascular system, cardiologists need to be involved in the diagnostic team. Countless studies unequivocally demonstrate that excess body weight is a significant risk factor for mortality and morbidity, including sudden cardiac death (SCD) [12]. Various underlying pathophysiological mechanisms contribute to this association. These include a higher prevalence of risk factors for cardiovascular diseases, as well as the presence of both electrical and structural ventricular remodeling, chronic inflammation, and an imbalance in the autonomic nervous system [11].

In our study, the prevalence of arrhythmias and other pathologies in 24-hour Holter monitoring in people with class 1 obesity and overweight was investigated. The analysis of the collected data indicates a significant increase in premature supraventricular beat (SPB) and premature ventricular beat (PVB), respectively, among participants with obesity (subgroup A) and participants who were overweight (subgroup B) when compared to the control group (subgroup C). PVB exhibited a strong correlation with body weight and both weight indexes. Moreover, our study revealed a positive linear correlation between body mass, SPB, and PVB. It is important to emphasize that while a linear correlation was observed between SPB and body weight, WHR, and BMI, in the case of PVB, this linear relationship was only observed according to body weight. The other types of arrhythmias occurred infrequently and were consistent with the prevalence in the normal weight control group.

Similar studies have been recently conducted by Skovgaard et al., and they included adults with overweight or obesity without cardiovascular diseases [13]. During a 24-hour Holter examination, they observed sinus tachycardia, which was present in 97% of participants, being the most common arrhythmia in this group. SPBs were present in 68% of patients, PVBs in 64% of patients, and sinus bradycardia in 34% of patients. There was a low prevalence of cases when PVBs were more than 200/24 h, only in 3% of the study group, and supraventricular premature beats were only generally presented without a detailed analysis. Ultimately, the authors claimed that these arrhythmias occur with the same frequency in people with normal weight, and additionally, no association between BMI and the occurrence of arrhythmias was found. However, the study has some flaws. First, it aims to analyze a healthy population with the strict inclusion criteria used during screening: the phase 1 clinical trial participants. It may be suggested that the authors’ policy was to select the proper group of participants with obesity or who were overweight for the forthcoming experiments. Moreover, it was commented that higher numbers of arrhythmias could be expected if less strict screening is applied. In this aspect, the data may not represent the frequency of abnormalities in an unselected population. In the abovementioned study, there was no division of the study group according to class of obesity, and it was mixed with overweight in statistical analysis. Eventually, the mean age was 36, compared to around 59.94 years in our study. 

In the study from 2016 by Hingorani et al. conducted on 1363 healthy subjects, 1000 males and 273 females, aged 18‣65 years, the authors found the general occurrence of SPBs at 60.8% and PVBs at 43,4%, when >200 PVBs per 24 h was detected in 3.3% patients. Supraventricular tachycardia was present in 2.2% of cases. Then, nonsustained ventricular tachycardia was present in 0.7% and second-degree AV block in 2.4% [14]. The group under study consisted of healthy volunteers in a clinical trial. The goal was like the abovementioned study of Sovgaard et al. from 2023; however, it was simultaneously not devoted to those with obesity and overweight [13]. Comparing the mentioned research studies with ours, it should be underlined that the unique feature of our study is the selection of random volunteers who were obese and overweight who suffered from some complications that are common in this group, including hypertension and diabetes mellitus. From this point of view, we present a more realistic survey of the arrhythmia prevalence in class 1 obesity and overweight. Moreover, we carried out a comparison of both subgroups, finding more pathologies in the class one obesity group than in overweight. Another study worth mentioning was conducted by Bienias et al., which included healthy young adults with class 3 obesity (BMI >40 kg/m2). The authors found no increased risk of arrhythmias in them compared to adults with normal weight. However, cardiac autonomic dysfunction was observed in morbidly obese participants through the assessment of heart rate variability and turbulence, which was impaired [15].

Some authors found a relationship between higher heart rate and obesity alone or co-existing with diabetes mellitus; however, in our study, we observed a higher prevalence of tachycardia in individuals with obesity, which did not reach statistical significance [16]. The explanation for the findings may be that the group was not numerous enough in our study, and first of all, our study group consisted of patients with obesity class 1 only. Moreover, some of our patients were taking beta blockers, which could have affected the occurrence of tachycardia in the studied population. In a recent study conducted by Binu et al., it was found that tachycardia is quite a common phenomenon in obese individuals (14.7%) and even more common in patients with morbid obesity (23.3%) [16]. 

The relationship between obesity and the sympathetic nervous system is still being discussed in the literature. Some studies have stated that increased sympathetic activity is an effect of obesity [17,18,19]. The review by Valensi describes sympathetic nervous system dysfunction leading to obesity, especially with visceral adiposity [20]. McCully et al.’s study showed, in mice, that autonomic dysfunction can affect excessive epicardial fat and lead to chronic inflammation and oxidative stress [21]. Moreover, people with obesity had higher heart rates than individuals with normal weight. The authors have explained it via the imbalance in the autonomic nervous system caused by a reduction in parasympathetic function in people with obesity [18,22]. Other studies have found that autonomic imbalance is connected to a higher risk of ventricular arrhythmia and sudden cardiac death in people who are obese [17,18]. It is also worth mentioning that in the study by Espinoza-Salinas et al., autonomic dysregulation was found both in adults who were overweight r had obesity [23].)

HRV is one method that can be used to evaluate sympathetic activity. In our study on analysis of the HRV time domain, only mRR in 24-hour monitoring and mRR during daily activity were significantly higher in patients who were obese and overweight than in the control group, and there was only a trend toward lower SDNN values in people with obesity. However, it did not reach the statistically significant level. In our study group, there were people only with class one obesity, and it is possible that if more individuals from class 2 and 3 were included, these differences would be significant. We also found no significant correlations between the study group’s body mass parameters and time domain heart rate variability parameters.

A study on a healthy Korean population found that body mass parameters were negatively associated with HRV parameters. However, the association of WHR and percentage of body fat mass were stronger indicators of HRV changes than BMI [24]. Another study including Taiwanese nurses found that nurses with higher waist circumferences had significantly lower power of low-frequency spectrum (LF), power of high-frequency spectrum (HF), and SDNN from time domain HRV analysis. BMI had a significant negative association with the power of very low-frequency spectrum (VLF) and SDNN [25]. Yadav et al.’s study found that increased WHR was strongly associated with reduced cardiac parasympathetic activity (measured as SDNN, RMSSD, NN50 count, pNN50, HF indices in milliseconds squared, and SD1) and increased sympathetic activity (LF/HF) in patients with obesity [22].

There are many other ways by which obesity might influence the pathophysiology of arrhythmia. They include metabolic disturbances, structural and electrical remodeling, neurohormonal adaptation, and associated diseases. Obesity is often associated with coexisting conditions like hypertension and obstructive sleep apnea. Hypertension leads to left ventricular hypertrophy (LVH) and can result in heart failure, which itself increases the prevalence of ventricular arrhythmia and SCD [26]. Valencia-Flores et al. found that patients who were morbidly obese with severe sleep apnea and oxygen saturation ≤65% had a higher risk of cardiac arrhythmias [27]. However, obesity may also lead to the development of atrial cardiomyopathy by affecting the hemodynamics of the heart [28]. 

Hemodynamic stress that comes from hypervolemia in patients with obesity increases cardiac output and leads to LVH [17,26]. Obesity causes an increase in pressure in the pulmonary circulation, resulting in right ventricular hypertrophy, which further leads to left ventricular hypertrophy. Subsequently, LVH contributes to left atrial dysfunction and hypertrophy, which may predispose to the development of AF. Fibrotic atrial cardiomyopathy (FACM) may contribute to AF and reduce the odds of spontaneous conversion to sinus rhythm [29]. Obesity also leads to chronic systemic inflammation, an important factor for FACM development and progression, because of the high metabolic activity of pro- and anti-inflammatory adipokines [17,29].

The high metabolic activity of adipocyte tissue leads to many metabolic disturbances. Some of the most important adipokines that should be mentioned are leptin, adiponectin, and ghrelin [11,26]. Leptin, primarily produced by white adipose tissue, significantly impacts various body functions [11,26]. High leptin levels were associated with LVH, increased myocardial fat volume, and cardiomyocyte hypertrophy [17]. It is also believed to stimulate the sympathetic component of the autonomous nervous system and be a factor in LVH inpatients with obesity [17,30,31]. The activity of the parasympathetic component is reduced, and the activity of the sympathetic component is increased, and as a result, both heart rate and blood pressure increase. Furthermore, leptin plays a crucial role in regulating the phenotype of fibroblasts, thus initiating a fibrogenic process [17].

A low adiponectin level, another important adipokine found in individuals with obesity, might result in LVH [26]. Adiponectin, a remarkable insulin-sensitizing adipokine, is produced solely by adipocytes. It is pivotal in enhancing insulin sensitivity, warding off arteriosclerosis, and reducing inflammation [17].

Neurohormonal adaptation associated with modification of the autonomic tone leads to a decrease in HRV. It also impacts the activation of the renin-angiotensin–aldosterone system, leading to high levels of Aldactone and, as a result, systolic and diastolic dysfunction, LVH, and myocardial fibrosis—another important factor of ventricular arrhythmia susceptibility [26]. Due to obesity, changes in cardiac cellular electrophysiology can lead to alterations in ion channels I_Na_, I_Ca,L_, and K_ATP_. In animal models of obesity, the expression of the inward potassium current clearly decreases and results in action potential prolongation, QTc prolongation, and a higher incidence of PVBs. Using a K_ATP_ channel opener reduced that effect, showing K _ATP_ channel inhibition in obese cardiomyocytes. Some studies also describe other proarrhythmic changes to calcium and sodium channels resulting in severe disturbance in calcium metabolism, which could lead to a higher incidence of ventricular arrhythmias [17]. 

Patel et al. suggested that obesity’s pro-arrhythmogenic role results from atrial remodeling and increased epicardial adipose tissue volume. Both were proven to cause changes in the heart’s conduction parameters, especially the P-wave parameters. Moreover, the role of comorbidities often coexisting with obesity was also highlighted as a possible factor leading to AF [4,32]. 

The literature documents the association between excessive body weight and AF. Several studies correlated a higher risk of AF with obesity, as the growing occurrence of transition from paroxysmal to persistent atrial fibrillation [11,32,33,34,35]. In our study, we observed an increase in the occurrence of AF among individuals with obesity or who were overweight compared to patients with normal weight. However, this observation did not reach statistical significance. Additionally, in patients with class 1 obesity, cases of atrial fibrillation and other serious arrhythmias were not frequent. There are also studies in the literature that commented on a relationship between weight loss and reduction in the incidence of AF [5,6,7].

PVBs and especially frequent PVBs (≥ one time during a standard electrocardiographic recording or ≥30 times over a 1-hour recording) were found to be linked with increased probability for SCD in patients with structural heart disease [26,36]. 

In the retrospective cohort study of cardiac MRI conducted by Wang et al., epicardial adipose tissue was linked to a higher incidence of PVBs and all-cause of mortality from SCD [26,37]. However, Wang’s study also revealed higher occurrences of tachyarrhythmia and atrial fibrillation, which was less observed in our cohort. The study mentioned above was conducted on 402 patients with PVBs, among whom 249 were at least overweight (defined as BMI ≥24 kg/m^2^). The control group consisted of 249 participants; in this group, 207 individuals were classified as having excessive weight. 

PVBs are predictors of numerous cardiovascular diseases. However, von Rotz et al.’s study investigated the occurrence of PVBs in healthy young adults, and it was found that PVBs are common even in populations without any cardiovascular comorbidities. PVBs occurred at least once in 68,7% of participants in their study. However, the number of PVBs depended on several risk factors, including increased WHR [38]. 

Sabbag et al.’s study found that individuals who were overweight and obese have a higher risk of occurrence of ectopic ventricular arrhythmia during exercise than individuals with normal weight [39]. The risk increased gradually with an increase in BMI. Moreover, the authors suggested that the increased risk of ventricular arrhythmias may predict a higher risk of sudden cardiac death in featured susceptible populations.

Our findings met statistical significance, but the question remains if they would be clinically significant. There is a need for further research into the long-term effects of those arrhythmia incidences and their correlations with AF and heart failure. Måneheim et al.’s study found an independent association between PVB recorded on 24-hour electrocardiogram monitoring and AF and heart failure, and this effect was altered by SPB frequency. The follow-up for that study was 17 years long and found that the coexistence of both PVBs and SPBs also accumulates the risk of occurrence of AF and/or HF, especially if PVBs were multiform [40]. Aizawa et al.’s study observed that some parameters of P-wave (P-wave durations greater than 130 ms), P-wave morphology, SPBs, PVBs, or runs could be associated with the significant risk of AF. However, the studies were not dedicated to people with obesity or who were overweight [41]. Our conclusions may indicate the adverse remodeling that, if given sufficient time, would eventually lead to clinically significant increases in hemodynamically impactful arrhythmia burden. Therefore, more studies should be performed on patients with class 2 and 3 obesity in the future.

The current study has some limitations. Firstly, the studied population was restricted to a relatively small sample group. Moreover, the participants came from only a small part of Poland, which means there is no ethnic diversity in the studied population. Due to this, the study results cannot be generalized to non-Caucasian people or Caucasian people from other world regions. Furthermore, we have not observed participants’ examination results for an extended period, so we were limited to single-day observations. The short observation period likely underestimates the number of arrhythmias occurring in the patients. Another limitation is that the study included only normal-weight, overweight, and class 1 obesity patients. Due to this, we could not observe the prevalence of arrhythmias in patients with class 2 and 3 obesity and compare it to the BMI groups included in the study.

Additionally, echocardiography was not performed at the screening visit as it was not feasible in this study. Potentially, it could provide new information. If the Holter monitoring had been extended to 48 h, more arrhythmias, especially atrial fibrillation, could have been identified. Furthermore, BMI is a measurement that only accounts for a person’s weight and height and does not differentiate between lean mass and fat mass. The study could have been improved by including additional bioelectrical impedance analysis (BIA) to consider body composition.

A similar study, with a larger group of participants, could provide more valuable data concerning arrhythmias in different BMI groups, including the influence of age, sex, and coexisting chronic diseases, e.g., hypertension. Furthermore, other populations also should be studied. A longitudinal study would be fascinating regarding the possibility of observing the incidence of arrhythmias with changing age, comorbidities, and BMI. In the age of wide usage of weight-loss medication, it seems crucial to study the relationship between body mass and its distribution and arrhythmias, not only AF but also other supraventricular and ventricular arrhythmias, and to use that knowledge in clinical use. Adopting such information into a more individual approach and patient screening would be valuable. Due to its ease of access and non-invasive nature, electrocardiography is a valuable tool in stratifying the risk of life-threatening arrhythmias. It would be beneficial to find markers that could predict SCD early in different BMI groups to gain a chance to prevent it. 

Clinicians should attempt to obtain the best knowledge on the prevalence of arrhythmia in those with obesity, as this group of patients is representative both in outpatients and in patients’ clinical conditions. It could be beneficial to identify the subgroups with higher risk. In our study, we have shown that in class one, obesity and overweight, only less severe pathologies may occur, and we suggest the necessity for more studies in this aspect.

## 5. Conclusions

Obesity is a complex disorder that causes compensatory cardiovascular changes, including electromechanical dysfunction, and one of its complications may be the incidence of various types of arrhythmias. In the current study, it has been revealed that in a group with class 1 obesity, no life-threatening arrhythmias were found, and there were very few cases of atrial fibrillation, supraventricular tachycardia, and ventricular tachycardia. The main observation from this study was that in patients with class 1 obesity, there is a higher number of premature supraventricular beats and premature ventricular beats than in patients with normal body weight. Additionally, higher body mass was positively correlated with the number of premature supraventricular and ventricular beats, and similar correlations were present between waist circumference and SPB. Since the number of people with obesity still increases and, simultaneously, global health is burdened by its consequences, it is necessary to continue research in the field evaluating the relationship between obesity and arrhythmias. Especially in further studies, more patients with class 2 and class 3 obesity should be included in the analysis of the co-existing disorders.

## Figures and Tables

**Table 1:** Clinical characteristics of the studied subgroups.

Parameter	Entire Study Group (*n =* 250)	Obesity (A, *n =* 98)	Overweight (B, *n =* 83)	Control Group (C, *n =* 69)	*p <* 0.05
Age (years)	59.94 ± 13.22	61.18 ± 11.07	53.40 ± 13.70	58.83 ± 15.33	ns
Gender (%/*n*)					
Male	41.6/104	50.0/49	50.6/42	18.8/13	A vs. C: 0.001
Female	58.4/146	50.0/49	49.4/41	81.2/56	B vs. C: 0.001
Height (cm)	167.37 ± 9.76	168.14 ± 9.65	168.64 ± 10.36	164.76 ± 8.78	ns
Weight (kg)	80.42 ± 17.50	95.09 ± 13.24	78.39 ± 11.10	62.02 ± 7.99	A vs. B: 0.001 A vs. C: 0.001 B vs. C: 0.001
BMI (kg/m^2^)	28.64 ± 4.99	33.62 ± 3.26	27.56 ± 1.34	22.86 ± 1.71	A vs. B: 0.001 A vs. C: 0.001 B vs. C: 0.001
Waist (cm)	95.99 ± 14.26	107.29 ± 10.16	95.29 ± 8.55	79.72 ± 7.55	A vs. B: 0.001 A vs. C: 0.001 B vs. C: 0.001
Hip circumference (cm)	106.60 ± 12.16	115.23 ± 7.32	104.26 ± 5.23	93.83 ± 14.14	A vs. B: 0.001 A vs. C: 0.001 B vs. C: 0.001
WHR	0.95 ± 0.74	0.93 ± 0.08	0.91 ± 0.09	1.05 ± 1.56	ns
Hypertension (%/*n*)	52.8/132	64.3/63	50.6/42	39.1/27	A vs. C: 0.001 B vs. C: 0.048
Myocardial infarction (%/*n*)	6.4/16	7.1/7	7.2/6	4.3/3	ns
Stroke (%/*n*)	2.8/7	2.0/2	3.6/3	2.9/2	ns
Atrial fibrillation (%/*n*)	8.8/22	8.2/8	12.0/10	5.8/4	ns
Deep vein thrombosis (%/*n*)	3.6/9	7.1/7	1.2/1	1.4/1	ns
Type 2 diabetes (%/*n*)	13.2/33	21.4/21	10.8/9	4.3/3	A vs. C: 0.002
Thyroid disease (%/*n*)	16.4/41	16.3/16	13.2/11	20.3/14	ns
Smoking (%/*n*)	13.2/33	9.2/9	15.8/13	15.9/11	ns

BMI—body mass index; WHR—waist–hip ratio; ns—not statistically significant (*p* > 0.05).

**Table 2:** Parameters of 24 h Holter ECG monitoring in the entire study group.

Parameters	Mean ± SD
HR min (bpm)	53.93 ± 0.50
HR max (bpm)	117.33 ± 1.19
HR mean (bpm)	72.73 ± 0.55
SPB	308.39 ± 88.75
PVB	374.10 ± 129.18
Bradycardia	27.94 ± 9.15
Bradycardia (bpm)	38.39 ± 4.37
Tachycardia	23.02 ± 12.70
Tachycardia (bpm)	149.16 ± 20.39
VT	0.11 ± 0.05
SVT	2.72 ± 1.38
AF (average number of episodes)	0.16 ± 0.12
Ventricular rhythm	0.06 ± 0.04

HR max—maximum heart rate; HR mean—average heart rate; HR min—minimum heart rate; SPB—premature supraventricular beat; PVB—premature ventricular beat; bradycardia min—minimum beats per minute; tachycardia max—maximum beats per minute; VT—ventricular tachycardia; SVT—supraventricular tachycardia; AF—atrial fibrillation.

**Table 3:** Parameters of time-domain heart rate variability (HRV) analysis in the entire study group.

Parameters	Mean ± SD
24 h monitoring (6:00–6:00)	
mRR (ms)	828.42 ± 103.06
SDNN (ms)	150.05 ± 49.14
rMSSD (ms)	36.11 ± 41.46
SDSD (ms)	26.65 ± 29.06
pNN50 (%)	8.88 ± 13.98
Daily activity (6:00–22:00)	
mRR (ms)	771.61 ± 102.59
SDNN (ms)	112.26 ± 38.55
rMSSD (ms)	31.79 ± 37.79
SDSD (ms)	23.37 ± 26.65
pNN50 (%)	7.02 ± 13.36
Night rest (22:00–6:00)	
mRR (ms)	960.74 ± 137.51
SDNN (ms)	107.57 ± 39.93
rMSSD (ms)	43.30 ± 52.70
SDSD (ms)	30.03 ± 34.42
pNN50 (%)	12.90 ± 17.38

mRR—mean RR interval during sinus rhythm; pNN50—NN50 count divided by the total number of all NN intervals; rMSSD—the square root of the mean of the sum of the squares of differences between adjacent NN intervals; SDNN—standard deviation of all NN intervals; SDSD—standard deviation of differences between adjacent NN intervals.

**Table 4:** Parameters of 24 h Holter ECG monitoring in the study subgroups.

Parameter	Obesity (A, *n =* 98)	Overweight (B, *n =* 83)	Control Group (C, *n =* 69)	*p <* 0.05
HR min (bpm)	54.32 ± 0.65	53.52 ± 0.73	53.87 ± 1.32	ns
HR max (bpm)	113.38 ± 1.71	118.20 ± 2.18	118.89 ± 2.31	ns
HR mean (bpm)	72.47 ± 0.85	72.17 ± 0.86	73.78 ± 1.19	ns
PVB	573.52 ± 218.17	171.18 ± 59.07	96.87 ± 23.30	A vs. C: 0.030
SPB	620.20 ± 301.46	359.19 ± 153.89	42.49 ± 6.18	A vs. C: 0.042
Bradycardia	29.96 ± 18.61	33.20 ± 15.18	18.74 ± 8.51	ns
Bradycardia (bpm)	38.00 ± 4.79	38.89 ± 4.38	38.27 ± 3.85	ns
Tachycardia	35.32 ± 31.04	19.42 ± 10.90	9.88 ± 3.10	ns
Tachycardia (bpm)	147.42 ± 19.10	151.36 ± 24.00	148.16 ± 17.42	ns
VT	0.15 ± 0.09	0.13 ± 0.10	0.03 ± 0.02	ns
SVT	1.17 ± 0.44	3.70 ± 3.21	3.76 ± 3.17	ns
AF (average number of episodes)	0.06 ± 0.04	0.41 ± 0.36	0.00 ± 0.00	ns
Ventricular rhythm	0.10 ± 0.10	0.06 ± 0.05	0.01 ± 0.01	ns

HR max—maximum heart rate; HR mean—average heart rate; HR min—minimum heart rate; ns—not statistically significant (*p* > 0.05); SPB—premature supraventricular beat; PVB—premature ventricular beat; bradycardia min—minimum beats per minute; tachycardia max—maximum beats per minute; VT—ventricular tachycardia; SVT—supraventricular tachycardia; AF—atrial fibrillation.

**Table 5:** Parameters of time-domain heart rate variability (HRV) analysis in the study subgroups.

Parameters	Obesity (A, *n =* 98)	Overweight (B, *n =* 83)	Control group (C, *n =* 69)	*p <* 0.05
24 h monitoring (6:00–6:00)				
mRR (ms)	815.11 ± 77.60	867.50 ± 120.08	785.49 ± 92.20	A vs. C: 0.046 B vs. C: 0.014
SDNN (ms)	147.10 ± 40.67	147.35 ± 58.91	159.47 ± 46.17	ns
rMSSD (ms)	42.79 ± 63.12	29.64 ± 11.89	35.77 ± 25.09	ns
SDSD (ms)	30.72 ± 42.97	21.70 ± 9.22	28.11 ± 21.78	ns
pNN50 (%)	10.59 ± 20.16	7.72 ± 7.49	7.98 ± 9.85	ns
Daily activity (6:00–22:00)				
mRR (ms)	759.95 ± 80.95	810.30 ± 109.98	726.55 ± 105.14	A vs. C: 0.045 B vs. C: 0.011
SDNN (ms)	113.56 ± 34.41	107.38 ± 41.11	118.22 ± 42.24	ns
rMSSD (ms)	36.86 ± 57.20	26.88 ± 11.02	31.54 ± 24.91	ns
SDSD (ms)	26.26 ± 38.80	19.62 ± 9.00	24.82 ± 22.02	ns
pNN50 (%)	8.61 ± 19.86	5.93 ± 6.32	6.20 ± 7.97	ns
Night rest (22:00–6:00)				
mRR (ms)	938.84 ± 102.87	1003.92 ± 168.56	925.27 ± 118.54	ns
SDNN (ms)	108.81 ± 44.16	101.56 ± 32.11	115.35 ± 45.24	ns
rMSSD (ms)	54.14 ± 80.10	34.82 ± 15.26	39.38 ± 30.72	ns
SDSD (ms)	36.93 ± 52.14	24.41 ± 10.78	27.88 ± 20.27	ns
pNN50 (%)	14.86 ± 22.46	11.83 ± 11.53	11.44 ± 16.67	ns

mRR—mean RR interval during sinus rhythm; ns—not statistically significant (*p* > 0.05); pNN50—NN50 count divided by the total number of all NN intervals; rMSSD—the square root of the mean of the sum of the squares of differences between adjacent NN intervals; SDNN—standard deviation of all NN intervals; SDSD—standard deviation of differences between adjacent NN intervals.

**Table 6:** Correlations between body mass parameters and 24-hour Holter ECG monitoring parameters in the entire study group.

Parameter	Body Weight (kg)	BMI (kg/m^2^)	Waist Circumference (cm)	Hip Circumference (cm)	WHR
HR min (bpm)	ns	ns	ns	0.30 (*p =* 0.010)	ns
HR max (bpm)	ns	ns	ns	ns	ns
HR mean (bpm)	ns	ns	ns	ns	ns
PVB	0.14 (*p =* 0.028)	ns	ns	ns	ns
SPB	0.14 (*p =* 0.028)	0.13 (*p =* 0.043)	0.14 (*p =* 0.031)	ns	ns
Bradycardia	ns	ns	ns	ns	ns
Bradycardia (bpm)	ns	ns	ns	ns	ns
Tachycardia	ns	ns	ns	ns	ns
Tachycardia (bpm)	ns	ns	ns	ns	ns
VT	ns	ns	ns	ns	ns
SVT	ns	ns	ns	ns	ns
AF	ns	ns	ns	ns	ns
Ventricular rhythm	ns	ns	ns	ns	ns

BMI—body mass index; WHR—waist–hip ratio; HR max—maximum heart rate; HR mean—average heart rate; HR min—minimum heart rate; ns—not statistically significant (*p* > 0.05); SPB—premature supraventricular beat; PVB—premature ventricular beat; bradycardia min—minimum beats per minute; tachycardia max—maximum beats per minute; VT—ventricular tachycardia; SVT—supraventricular tachycardia; AF—atrial fibrillation.

**Table 7:** Backward stepwise multiple regression models in the entire study group for SPB as the dependent variable.

Model for: SPB			
	**BMI (kg/m^2^)**	**Type 2 Diabetes**	**Thyroid Disease**
Regression coefficient (RC)	82.292	791.956	918.975
SEM of Rc	39.721	384.861	352.687
*p*	0.037	0.040	<0.025
*p* for the model	0.001		

## Data Availability

The data are not publicly available due to patients’ privacy.
